# Diagnostic value of multiple b-value diffusion-weighted imaging in discriminating the malignant from benign breast lesions

**DOI:** 10.1186/s12880-022-00950-y

**Published:** 2023-01-11

**Authors:** Chu-Xin Lin, Ye Tian, Jia-Min Li, Shu-Ting Liao, Yu-Tao Liu, Run-Gen Zhan, Zhong-Li Du, Xiang-Rong Yu

**Affiliations:** Department of Radiology, Zhuhai Hospital Affiliated With Jinan University (Zhuhai People’s Hospital), 79 Kangning Road, Zhuhai, 519000 People’s Republic of China

**Keywords:** Breast Cancer, Multiple b-value diffusion-weighted imaging, Intravovel incoherent motion, Stretched-exponential model, Aquaporins

## Abstract

**Objective:**

The conventional breast Diffusion-weighted imaging (DWI) was subtly influenced by microcirculation owing to the insufficient selection of the b values. However, the multiparameter derived from multiple b-value exhibits more reliable image quality and maximize the diagnostic accuracy. We aim to evaluate the diagnostic performance of stand-alone parameter or in combination with multiparameter derived from multiple b-value DWI in differentiating malignant from benign breast lesions.

**Methods:**

A total of forty-one patients diagnosed with benign breast tumor and thirty-eight patients with malignant breast tumor underwent DWI using thirteen b values and other MRI functional sequence at 3.0 T magnetic resonance. Data were accepted mono-exponential, bi-exponential, stretched-exponential, aquaporins (AQP) model analysis. A receiver operating characteristic curve (ROC) was used to evaluate the diagnostic performance of quantitative parameter or multiparametric combination. The Youden index, sensitivity and specificity were used to assess the optimal diagnostic model. T-test, logistic regression analysis, and Z-test were used. *P* value < 0.05 was considered statistically significant.

**Result:**

The ADC_avg_, ADC_max_, f, and α value of the malignant group were lower than the benign group, while the ADC_fast_ value was higher instead. The ADC_min_, ADC_slow_, DDC and ADC_AQP_ showed no statistical significance. The combination (ADC_avg_-ADC_fast_) yielded the largest area under curve (AUC = 0.807) with sensitivity (68.42%), specificity (87.8%) and highest Youden index, indicating that multiparametric combination (ADC_avg_-ADC_fast_) was validated to be a useful model in differentiating the benign from breast malignant lesion.

**Conclusion:**

The current study based on the multiple b-value diffusion model demonstrated quantitatively multiparametric combination (ADC_avg-_ADC_fast_) exhibited the optimal diagnostic efficacy to differentiate malignant from benign breast lesions, suggesting that multiparameter would be a promising non-invasiveness to diagnose breast lesions.

## Introduction

The risk of cancer diagnosis has increased globally, and breast cancer remains the most frequently diagnosed female cancer that takes up almost 23% of the whole positive cases [1]. The breast cancer mortality rate shows a significant increase in 25 years throughout the world, increasing tendency in incidence and prevalence of this cancer account for the high mortality rate. Owing to its highly heterogeneous and metastatic character, it could commonly metastasize to distant organs, which was directly responsible for its malignance. Previous studies have reported that the 5-year survival rate of breast cancer patients is over 80% owing to the timely detection of this disease in North American [2]. Therefore, the early diagnosis in discriminating the benign breast lesions from malignancy was of considerable importance, which was helpful to its clinical treatment, good prognosis as well as increased survival rate.

Mammography was used as a convenient mean to screen the early-stage breast cancer. However, the dense breast tissue possibly obscured the potential mass in symptomatic women, resulting in the false-negative rate ranging from 8 to 66% [3, 4]. Some previous studies focused on the short acquisition time of ultrasound (US) imaging. However, it is seldom clinically used to evaluate the dynamic information of a contrast agent in lesions [5–7]. Nevertheless, even though magnetic resonance imaging (MRI) or Dynamic Contrast Enhanced MRI (DCE-MRI) technique could generate superior results to analyze the breast lesions and compensate for its insufficiency of ultrasound examination, abundant in the spatial and temporal variation, the disadvantages of DCE-MRI could not be neglected [8]. The nephrogenic fibrosing dermopathy was correlated to the Gd-DTPA that has been extensively used worldwide in MRI evaluation as a component of intravenously administered contrast agents [9]. Some patients who received intravenous injection may show severe anaphylactoid reaction [10]. Moreover, the ongoing discussion about contrast agents intravenous exposure associated with neuronal tissue deposition even in the setting of relatively normal renal function and its deposition in the brain additionally highlights the need for alternative [11].

Nowadays, diffusion-weighted MRI (DWI) has been recognized as an attractive non-invasive, quantitative, adjuvant technique reflecting the functional information about the Brownian motion of water molecules as well as the microscopic organization and cellularity of biologic tissues [12]. Numerous evidence has found that DWI can address some limitations of conventional breast MRI by offering complementary information with short acquisition time and highly diagnostic sensitivity for lesions assessment. The ADC value would be used as an effective parameter to distinguish between malignant and benign breast lesions [13, 14]. Whereas the monoexponential model applied to conventional DWI was limited by a single b value and the threshold obtained to distinguish malignancy varied from person to person [15, 16], which led to the bias that the dispersive result could partly influenced by tissue perfusion. The emergence of the biexponential model-intravovel incoherent motion (IVIM) proposed by Le Bihan et al. contributed significantly to improving the effectiveness of microcirculation, the microscopic blood flow in tissue with rich perfusion [17]. IVIM technique can separate microvessel perfusion from diffusion by setting a range of b value. The quantitative parameters included microvascular volume fraction (f), molecular diffusion coefficient (ADC_slow_) and perfusion-related incoherent microcirculation (ADC_fast_). ADC_slow_ represents the mobility of water molecules in tissue and depends on the cellularity, tortuosity of the extracellular space, integrity of cell membranes, and viscosity of fluids, f reflects the relative contribution of microvascular blood flow to the DWI signal, and ADC_fast_ depends on blood velocity and length of microvessel segments [18]. To tackle the limitation of the hypothesis of two diffusion compartments [19], Bennett et al. proposed a stretched-exponential model as supplementary and it has been widely applied to the brain, liver and prostate but rarely in the breast [20, 21]. The distribution diffusion coefficient (DDC) and the heterogeneity index (α) were used to describe the behavior of signal attenuation analytically as a function of b values. The DDC derived from the fitting stretched-exponential function to the data was associated with ADC_slow_ [22]. Moreover, with the effect of ultra-high b-value, aquaporins (AQP) reflecting physiologically osmotic water transport across cell plasma membranes at cellular level, facilitating transepithelial fluid transport and its expression became more sensitive to the change of functional b values [23, 24]. Mostly research focused on the different subtypes of AQP expression in vivo [25], however, there was a lack of studies on ADC_AQP_ as a biomarker differently expressed for breast cancer prediction [26]. Therefore, various elements should be considered when distinguishing the doubtful breast lesion.

Our study aimed to evaluate whether multi-b-values of DWI parameters could provide more useful information to an optimal diagnostic fitting model capable of accurately differentiating the benign breast lesions from malignancy and in-depth explored multiparametric combination as a credible non-invasive examination was beneficial to decrease the unnecessary risk of biopsies.

## Materials and methods

### Patients

This prospective study was approved by our institutional review board and written informed consent was waived. From April 2018 to August 2020, a total of 79 patients clinically diagnosed with breast lesions were included in the present study, among them, 38 patients suffered malignant breast tumors, and the remainder was diagnosed with benign lesions. The inclusion criteria were as follows: (a) All lesions underwent pathological confirmation, (b) MRI was performed using 3.0 T magnet, (c) The conventional sequence, single b-value DWI and multiple b-value DWI were all performed, (d) Patient had not undergone prior hormonal, chemotherapy, radiation treatment or any other neoadjuvant systemic treatment. (e) The patient went through at least 2 years of follow-up. (f) Only the largest mass lesion confirmed by histopathology in each patient was chosen for detailed analysis. The clinical data, such as age, menopause stage, carcinoembryonic antigen (CEA), carbohydrate antigen 125 (CA125), CA153, and CA199 were obtained from patients.

### MRI scans

All MR examinations were performed in the prone position using a 3 T MR scanner (Discovery MR 750, GE Healthcare) with an 8-channel breast coil in the prone position. Following axial turbo spin-echo T_1_-weighted imaging (repetition time[TR]/ echo time[TE], 95.6 ms/minimum; field of view[FOV],32 × 32cm^2^; slice thickness,4 mm;spacing,0 mm; number of excitation[NEX], 1;slices,28; time,1min13sec),the axial turbo spin-echo T_2_-weighted fat-suppressed imaging (TR/TE,5096/87.8 ms; FOV,32 × 32cm^2^; slice thickness,4 mm; spacing,1 mm; NEX,3; slices,28; time, 1min07sec) and the sagittal T_2_-weight imaging obtained using fast-field -echoes with fat-saturated (TR/TE,2950/92.1 ms; FOV,20 × 20; slice thickness, 4 mm; NEX, 2; slices, 21; spacing,1 mm; time,1min52s) were performed successively. Multi-b-value DWI was also performed using single-shot echo planar and water excitation fat saturation imaging (TR/TE, 2000/97.7 ms; FOV, 36 × 36cm^2^; slice thickness, 4 mm; spacing,1 mm; slices,28; time,8 min; b values, 0, 30, 50, 70, 100, 150, 200, 400,600, 1000, 1500, 2000, 3000 s/mm^2^; NEX, 2, 2, 2, 2, 2, 2, 2, 3, 3, 3, 4, 4 and 6 respectively, temporal resolution,1 min 1 s/phase). Conventional and multiple-b value DWI were conducted before the injection of contrast agents. Axial T_1_-weighted DCE MRI images were acquired using volume image breast assessment (VIBRANT) gradient-echo sequence with the following parameters: TR/TE, 4.7/2.2 s; FOV,36 × 32.4; slice thickness,2 mm; spacing,0 mm; slices,128; time,45 s; NEX,0.71. The axial DEC-MRI images were obtained with the same imaging parameters (except for the time, 8min31s).

### Image analysis

Post-processing was performed using monoexponential, biexponential, stretched-exponential and ADC_AQP_ model were analyzed by GE Discovery MR 750 3.0 T to generate diffusion parameters and calibrated parametric breast maps. The parameters (ADC_min_, ADC_avg,_ ADC_max,_ f, ADC_fast_, ADC_slow_, DDC, α, and ADC_AQP_) originated from relative models were well matched. Under the guideline of the T2WI and DCE MRI image, the region of interest (ROI) was placed on the axial DWI images with a b-value of 1000 s/mm^2^ and the largest tumor transverse-sectional level around the edge of the lesion was away from the partial volume effect, cystic, calcific and necrotic areas as much as possible. The relevant formulas are applied as follows:

1. monoexponetial diffusion model [17]:
  1 $${\text{S}}\left( {\text{b}} \right){\mkern 1mu} /{\text{ S}}\left( 0 \right){\mkern 1mu} = {\text{exp}}^{{( - {\text{b}}^{*} {\text{ADC}})}}$$
  where S(b) and S(0) represent the signal intensity of b-values of b and 0 severally. The ADC stands for apparent diffusion coefficient.
2. biexponential diffusion model [27]:
  2 $${\text{S}}\left( {\text{b}} \right){\text{ }}/{\text{ S}}\left( 0 \right) = {\text{ f}}^{*} \exp ^{{( - {\text{b}}^{*} {\text{ADC}}_{{{\text{fast}}}} )}} + \left( {1 - {\text{f}}} \right)^{*} \exp ^{{( - {\text{b}}^{*} {\text{ADC}}_{{{\text{slow}}}} )}}$$
  where ADC_fast_, ADC_slow_ as well as f denote the true diffusion coefficient, pseudodiffusion coefficient and the fraction of perfusion.
3. stretched-exponential diffusion model [22]
  3 $${\text{S}}\left( {\text{b}} \right)/{\text{S}}\left( 0 \right) \, = exp^{{\left( { - \left( {b^{*} DDC} \right)^{\alpha } } \right)}}$$
  The distributed diffusion coefficient (DDC) and the water molecular diffusion heterogeneity index (α) were standard parameters in stretched-exponential model. DDC reflect the mean intravoxel diffusion rate and α ranging from 0 to 1 is associated with the inrtavoxel water molecular diffusion heterogeneity.
4. ADC_AQP_ images were generated with the pixel-wise mono-exponential interpolation of ultra-high b-value DWI images according to Eq. (i) by using the AQP module build-in Functional Tool of workstation.
  4 $${\text{S}}\left( {\text{b}} \right){\text{ }}/{\text{ S}}\left( 0 \right){\text{ }} = \exp ^{{( - {\text{b}}^{*} {\text{ADC}}_{{{\text{AQP}}}} )}}$$

### Histopathologic analysis

All specimens were pathologically confirmed with breast lesions in two weeks. During follow-up, they were analyzed retrospectively by an experienced pathologist who was unaware of the MRI outcomes and corresponding clinical information. The quantitative parameters of multiple b-values were measured by two radiologists with at least 10-year experience blind to the histopathological and clinical information. Besides, the conventional MRI characteristics evaluated by them conclude tumor maximum diameter, tumor position (upper-inner quadrant, upper-outer quadrant, lower-inner quadrant, and lower-outer quadrant), internal enhancement pattern (homogeneous, heterogeneous, rim enhancement), the delayed phase of time-intensity curve (TIC) [persist(I), plateau (II), and washout (III)], and breast density (fatty, fibro-glandular, heterogeneously dense, and extremely dense). In case of discrepancy on such above categorical variables, the judgement of the third doctor with higher qualifications prevails.

### Statistical analysis

An intra-class correlation coefficient (ICC) was calculated to evaluated interobserver reliability of quantitative DWI multiparameter measurement. The Shapiro–Wilk test was used to test the measurement data normal distribution. Categorical variables were presented as numbers and percentage. Continuous variables were presented as means and standard deviations or median and interquartile rages depending on its distribution. Categoric data were calculated using chi-square test of Fisher’s exact test. Continues data were calculated using Mann–Whitney U test or Student t-test. Diagnostic performance was evaluated using the area under the receiver operating characteristic curves (AUCs), standard error (SE) analysis sensitivity, specificity and 95% confidence interval. The analysis was performed both on the single variates and on a combination of multiple parameters as well. Based on histopathologic results as the gold standard. Youden indices were employed for defining cut-off value. Z test was used to compare AUCs between samples and populations for diagnostic accuracy. In all case, statistical significance was accepted with a *P* value of 0.05.

## Results

A total of 79 lesions were confirmed by pathological examination clinically. The 38 patients (median, 48.5 years; range, 42.25–56.50 years) with breast cancer comprised 21 invasive ductal carcinoma (IDC) cases, 1 ductal carcinoma in suit (DCIS) case, 1 squamous carcinoma case and the remaining 15 cases were invasive ductal carcinomas with carcinoma in suit. While benign group (median 47 years; range, 38–50 years) classifications consisted of breast 20 fibroadenomas cases, 16 cystic hyperplasias cases, and 5 adenosis of mammary cases.

**Fig. 1:**
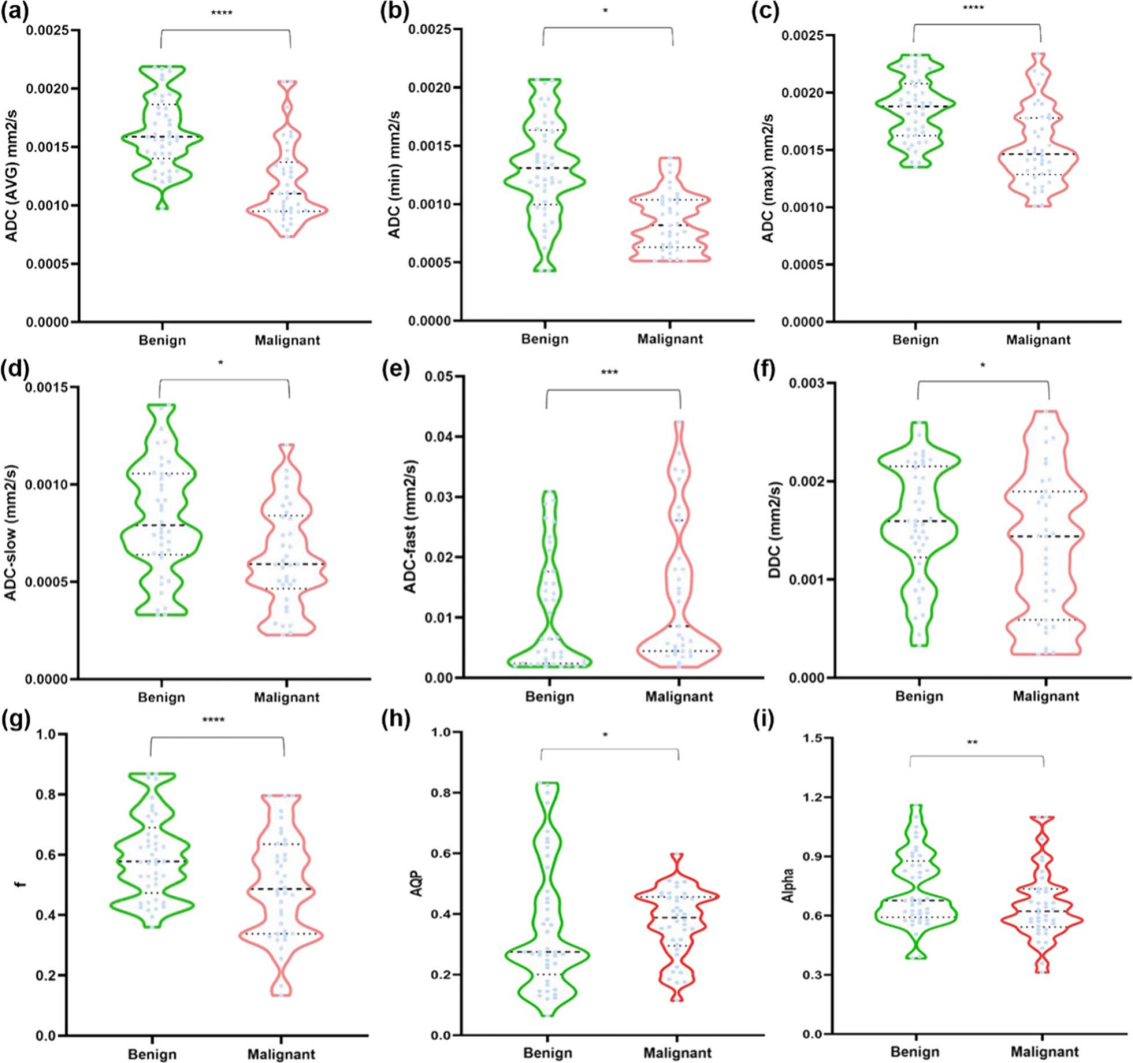
The box plots of ADC_avg_ (**a**), ADC_min_ (**b**), ADC_max_ (**c**), ADC_slow_ (**d**), ADC_fast_ (**e**), DDC (**f**), f (**g**), ADC_AQP_ (**h**) and α (**i**) values between benign and malignant tumors derived from mono-exponential, bi-exponential, stretched exponential diffusion model and AQP exponential model

The comparison of clinical and conventional imaging characteristics between benign and malignant groups was shown on Table 1. It reveals that the clinical and some conventional imaging characteristics show no significance. The quantitative parameters derived from multiple b-value measurement data underwent Shapiro–Wilk test and satisfy normal distribution or nearly normal distribution (P > 0.05). The intraclass correlation coefficient (ICCs) of all parameters were raging from 0.801 to 0.964, which represented the measurements of multiparameter had a good interobserver reproducibility. Details were shown in Table 2. Table 3 showed the mean difference between malignant and benign groups. The ADC_avg_ (0.016 ± 0.0003 vs. 0.013 ± 0.0005), ADC_max_ (0.0019 ± 0.0003 vs. 0.0015 ± 0.0003) and α (0.7317 ± 0.1875 vs. 0.6497 ± 0.1767) values of benign lesion group were larger than those of the malignant lesions group (*P* < 0.05).While the ADC_fast_ value of the benign group was significantly lower than the malignant measurement (0.0133 ± 0.0158 vs. 0.0428 ± 0.0741) (*P* < 0.05). Conversely, the f value of benign tumor obviously larger (0.05940 ± 0.1410 vs. 0.4948 ± 0.1744) (*P* < 0.05). The ADC_AQP_, ADC_min_, ADC_slow_ and DDC values within two groups exhibited no statistical significances (*P* > 0.05). The box charts based on the analytical result above were portrayed in Fig. 1 and directly reflected the distribution of each parameter between benign and malignant lesions, offering valuable reference information.

**Table 1:** Comparison of clinical and conventional imaging characteristics between benign and malignant groups

Variables	Benign (n = 41)	Malignant (n = 38)	*p*
Age	47.00 [38.00, 50.00]	48.50 [42.25, 56.50]	0.159
CA153	8.00 [5.50, 9.90]	9.05 [5.23, 12.85]	0.301
CA125	7.70 [5.30, 13.10]	6.70 [5.58, 12.30]	0.495
CA199	14.23 [8.60, 22.37]	13.86 [8.80, 20.87]	0.549
CEA	1.00 [0.51, 1.82]	0.85 [0.47, 1.94]	0.768
Tumor longest diameter	14.40 [10.60, 17.50]	15.40 [13.43, 20.82]	0.116
*Menopause stage*			0.203
Post	6 (14.63)	11 (28.95)	
Pre	35 (85.37)	27 (71.05)	
*internal enhancement pattern*			0.112
Homogeneous	27 (65.85)	23 (60.53)	
Heterogeneous	5 (12.20)	11 (28.95)	
Rim enhancement	9 (21.95)	4 (10.53)	
*TIC*			0.428
I	13 (31.71)	13 (34.21)	
II	15 (36.59)	9 (23.68)	
III	13 (31.71)	16 (42.11)	
*Tumor position*			0.163
Upper-inner quadrant	9 (21.95)	12 (31.58)	
Lower-inner quadrant	8 (19.51)	4 (10.53)	
Upper-outer quadrant	15 (36.59)	19 (50.00)	
Lower-outer quadrant	9 (21.95)	3 (7.89)	
*Breast dense*			0.970
Fat	1 (2.44)	1 (2.63)	
Fibro-glandular tissue	6 (14.63)	7 (18.42)	
Heterogeneous fibro-glandular tissue	20 (48.78)	17 (44.74)	
Extreme fibro-glandular tissue	14 (34.15)	13 (34.21)	

TIC represents time-intensity curve and persist(I), plateau (II), and washout (III). CEA represents carcinoembryonic antigen, CA 125 represents carbohydrate antigen 125, CA153 represents carbohydrate antigen 153, CA199 represents carbohydrate antigen 199

**Table 2:** Interobserver consistency of Interobserver consistency of multiparameter derived from respective model

	ICC	95% confidence interval
ADC_avg_ (× 10^−3^mm^2^/s)	0.842	0.764–0.896
ADC_fast_(× 10^−3^mm^2^/s)	0.964	0.944–0.977
ADC_max_ (× 10^−3^mm^2^/s)	0.801	0.706–0.868
ADC_min_ (× 10^−3^mm^2^/s)	0.957	0.934–0.972
ADC_slow_ (× 10^−3^mm^2^/s)	0.963	0.943–0.976
f	0.949	0.921–0.967
DDC (× 10^−3^mm^2^/s)	0.959	0.936–0.973
$\alpha$	0.954	0.930–0.971
ADC_AQP_(× 10^−3^mm^2^/s)	0.947	0.919–0.966

*ICC* Intraclass correlation coefficient

**Table 3:** Mean difference of patients with benign and malignant breast tumor respectively

Variables	Mean ± SD		Mean Difference	*P* value
	Malig = 0 (n = 41)	Malig = 1 (n = 38)		
ADC_avg_	0.0016 ± 0.0003	0.0013 ± 0.0005	0.0004	0.0000
ADC_min_	0.0013 ± 0.0004	0.0011 ± 0.0014	0.0002	0.4140
ADC_max_	0.0019 ± 0.0003	0.0015 ± 0.0003	0.0003	0.0000
ADC_slow_	0.0012 ± 0.0014	0.0008 ± 0.0007	0.0003	0.1830
ADC_fast_	0.0133 ± 0.0158	0.0428 ± 0.0741	-0.0294	0.0150
DDC	0.0016 ± 0.0006	0.0019 ± 0.0027	-0.0003	0.4650
f	0.5964 ± 0.1382	0.4948 ± 0.1744	0.0994	0.0060
α	0.7317 ± 0.1875	0.6497 ± 0.1767	0.0820	0.0490
ADC_AQP_	0.3636 ± 0.2175	0.3699 ± 0.1139	-0.0063	0.0874

“0” represents benign group; “1”represents malignant group; *SD* Standard differences. Comparisons were performed by independent t test

**Fig. 2:**
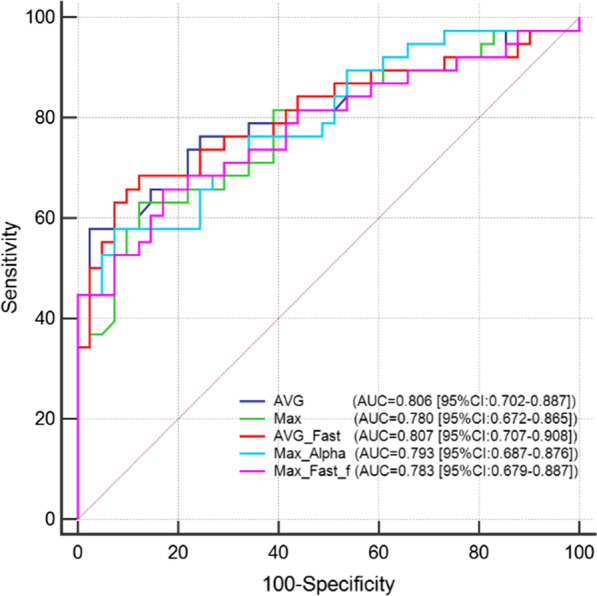
The ROC and AUC analysis of ADC_avg_, ADC_max_, ADC_avg-_ADC_fast_, ADC _max-_α and ADC_max_-ADC_fast_-f derived from multiple b values models for differentiation between breast cancer and benign lesions regions of interest

**Fig. 3:**
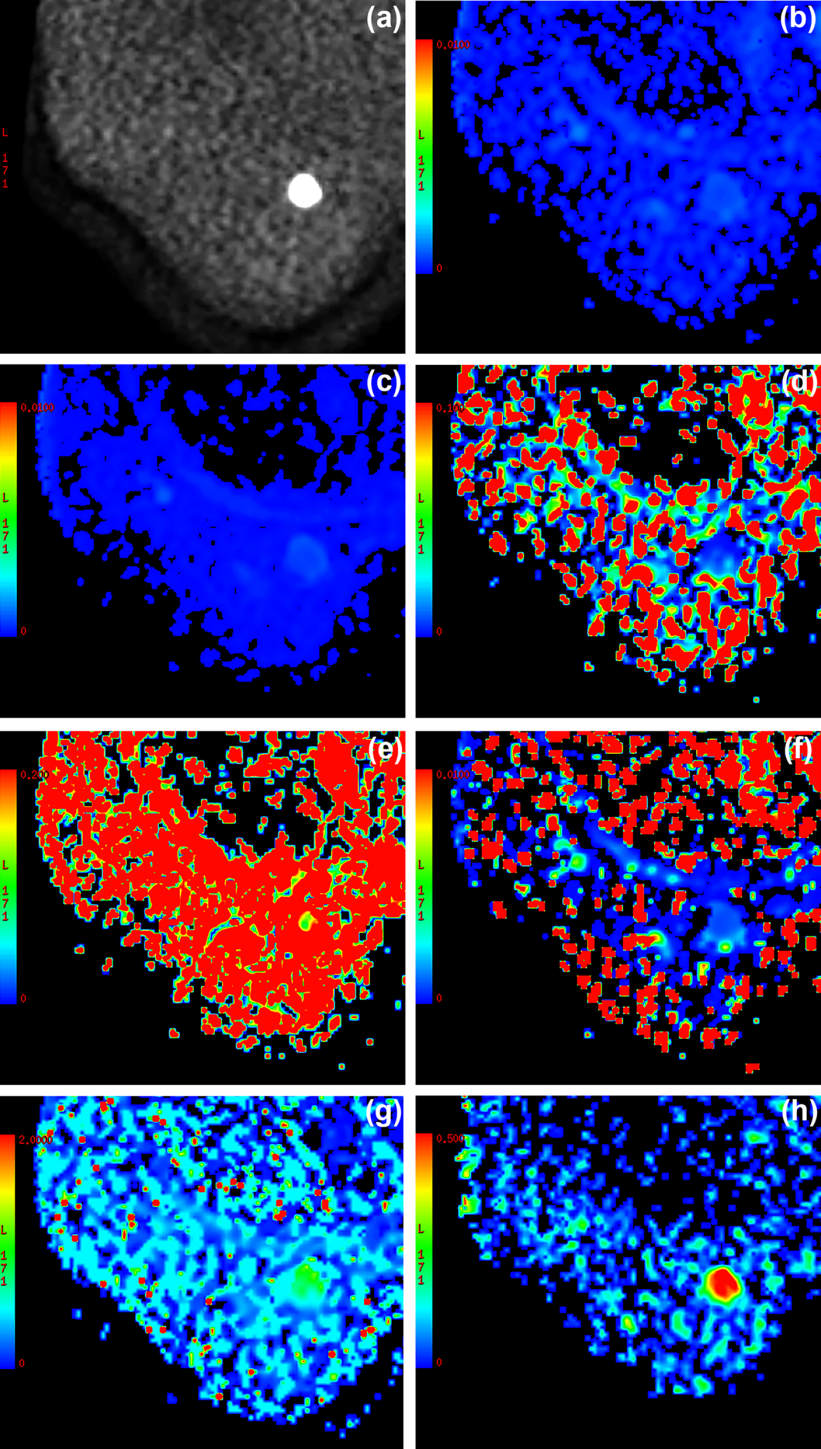
A 61-year-old patient was pathologically confirmed with malignant breast invasive ductal carcinoma on the left breast correlating area, measured 33 mm $\times$ 26 mm. DWI image with b-value of 1000 s/mm^2^ (**a**) shows the ROI on the whole tumor and displays the ROI at tumor margin. Parametric maps of (**b**) average standard ADC: 0.68 × 10^−3^mm^2^/s; (**c**) ADC_slow_: 0.62 × 10^−3^mm^2^/s; (**d**) ADC_fast_: 6.97 × 10^−3^mm^2^/s; (**e**) f: 0.195; (**f**) DDC: 0.59 × 10^−3^mm^2^/s; (**g**) α: 0.727 and (**h**) ADC_AQP_: 0.508um^2^/ms were demonstrated, respectively

**Fig. 4:**
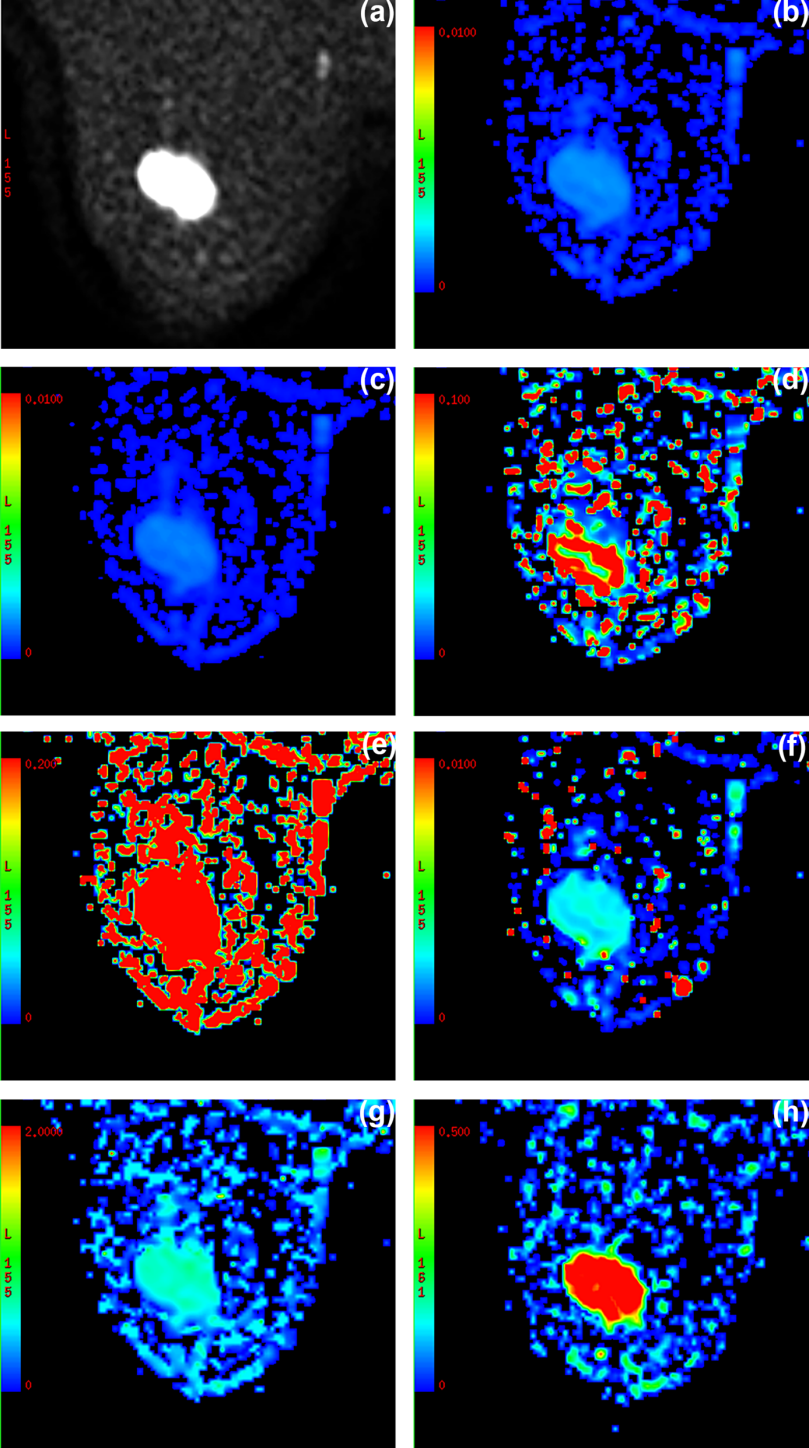
A 48-year-old patient who was pathologically confirmed with benign breast fibroadenomas exhibited a mass on the left breast in the correlating area, measured 10 mm $\times$ 13 mm. Its region of interest (ROI) was based on (**a**) axial STIR-DWI at b-value = 1000 s/mm^2^ with hyperintensity. Parametric maps of (**b**) average standard ADC: 1.39 × 10^−3^mm^2^/s; (**c**) ADC_slow_: 1.10 × 10^−3^mm^2^/s; (**d**) ADC_fast_: 6.14 × 10^−3^mm^2^/s; (**e**) f: 0.326; (**f**) DDC: 2.56 × 10^−3^mm^2^/s; (**g**) α: 0.596 and (**h**) ADC_AQP_: 0.604um^2^/ms were demonstrated, respectively

The pathological results of breast lesions were recognized as the gold standard. Multiparametric combination of monoexponetial, biexponetial and stretched exponential parameters based on the statistical difference were summarized in Table 4. Interestingly, observing the combined AUC of the two parameters (ADC_avg_-ADC_fast_) (0.807; 95% confidence interval, 0.707–0.901) (*P* < 0.05) identified with the AUC of three parameters (ADC_avg-_ADC_fast_-α) (0.807; 95% confidence interval, 0.703–0.887) displayed the largest AUC and both of them were considerably greater than that of each parameter alone. Nevertheless, the Youden index of ADC_avg_-ADC_fast_ was higher than that of ADC_avg-_ADC_fast_-α, maintaining the specificity of 87.8% and sensitivity of 68.42%. On the basis, this position would be determined as the cut-off point. Then, the AUC of ADC_avg_ (0.806, 95% confidence interval, 0.702–0.887) closely followed. The predictive values of the other multiparametric combinations or independent parameter, with AUCs below 0.80. Besides, ADC_avg-max_-α was the best specificity with the highest average of 92.68% but lowest sensitivity of 55.26% on the contrary. The ROC curves of the mono-exponential, bi-exponential and stretched-exponential parameters were drawn in Fig. 2, all of which was selected with good diagnostic performance (AUC at least for 0.75) and compared with parameter involved alone. The result ultimately revealed that the diagnostic efficacy of combination of ADC_avg-_ADC_fast,_ ADC_max_-α, ADC_max_-ADC_fast_-f were markedly greater (*P* < 0.05). Figures 3 and 4 provided functional parameter maps of benign and malignant breast lesions.

**Table 4:** Area under the curve (AUC) and relevant parameters of the monoexponential, biexponential, stretched exponential diffusion model, and multiparametric combination

Variable	AUC	SE	95%CI	z statistic	Youden index	Sensitivity	Specificity
ADCavg	0.806	0.051	0.702–0.887	5.934	0.5546	57.89	97.56
ADCmax	0.780	0.053	0.672–0.865	5.242	0.5096	63.16	87.80
ADCfast	0.674	0.061	0.559–0.775	2.868	0.3132	94.74	36.59
f	0.661	0.062	0.546–0.764	2.581	0.3440	36.84	97.56
α	0.634	0.063	0.519–0.740	2.134	0.2567	84.21	41.46
ADCavg-max	0.794	0.051	0.694–0.894	5.721	0.4814	57.89	90.24
ADCavg-fast	0.807	0.051	0.707–0.908	5.941	0.5623	68.42	87.80
ADCavg-f	0.791	0.052	0.689–0.894	5.517	0.5019	52.63	97.56
ADCmax-fast	0.776	0.054	0.670–0.882	5.080	0.4872	65.79	82.93
ADCmax-f	0.777	0.053	0.673–0.882	5.169	0.5096	63.16	87.80
ADCmax-α	0.793	0.051	0.687–0.876	5.755	0.5058	57.89	92.68
ADCfast-f	0.673	0.061	0.553–0.793	2.803	0.3498	44.74	90.24
ADCfast-α	0.666	0.061	0.551–0.768	2.733	0.2696	68.42	58.54
ADCavg-max-fast	0.791	0.052	0.690–0.893	5.595	0.5077	60.53	90.24
ADCavg-max-f	0.789	0.052	0.688–0.890	5.570	0.4852	63.16	85.37
ADCavg-max-α	0.795	0.051	0.689–0.877	5.787	0.4795	55.26	92.68
ADCavg-fast-f	0.798	0.052	0.697–0.900	5.704	0.5340	63.16	90.24
ADCavg-fast-α	0.807	0.051	0.703–0.887	5.972	0.5603	65.79	90.24
ADCavg-f-α	0.798	0.052	0.692–0.880	5.745	0.5077	60.53	90.24
ADCmax-fast-f	0.783	0.053	0.679–0.887	5.303	0.4872	65.79	82.93
ADCmax-fast-α	0.792	0.051	0.686–0.875	5.697	0.4833	60.53	87.80
ADCmax-f-α	0.792	0.051	0.686–0.875	5.709	0.5058	57.89	92.68
ADCfast-f-α	0.697	0.059	0.583–0.795	3.325	0.3293	50.00	82.93

*AUC* Area under the curve, *SD* Standard difference, *SE* Standard error, *CI* Confidence interval

## Discussion

Diffusion-weighted MRI has currently been incorporated into breast MRI protocols to improve the sensitivity of potential cancer whose ADC value has been considered as a quantitative index to address some deficiencies of routine clinical breast MRI. Compared with other detective techniques, the non-contrast and predictive assessment of differentiation of benign and malignant breast lesion showed superiority [13]. Mebis et al. [28] explored the correlation between average and minimum ADC values on MRI and they both showed significant differences in disease classification. To investigate the possible influence on microcirculation to molecular diffusion motion, we took ADC_avg_, ADC_min_ and ADC_max_ value of monoexponential diffusion model into consideration. The result demonstrated that it was the ADC_avg_ value that had an AUC of 0.806 and the better Youden index with a sensitivity of 57.89% and specificity of 97.56%, indicating that ADC_avg_ value was a reliable quantitative measurement for predicting malignant breast lesions preoperatively. Nonetheless, it deviated from the previous research conducted by Rupa R et al. who found that the minimum ADC value was reported to be more accurate in classifying the grade of breast tumor than mean ADC value [29]. The variation revealed that clear consensus had yet been reached regarding this association, suggesting that the perfusion was somehow likely to have impact on the ADC value.

Multiparametric MRI diffusion models have been currently served as a feasible technique to characterize quantitatively of lesions, such as liver fibrosis, prostate cancer, and salivary gland lesions [30–32]. The ADC_slow_, ADC_fast_ value and f derived from biexponential model represented tissue true diffusivity, perfusion-related incoherent microcirculation along with perfusion fraction respectively while even though ADC_slow_ value (0.0012 ± 0.0014 vs. 0.0008 ± 0.0007, *P *= 0.1830) of IVIM manifested increasing cellularity and confined extracellular spaces of breast cancer, it showed no statistical difference between benign and malignant lesions, which was inconsistent with the previous study [32]. Perhaps the rough definition failed to sort out the mean or 50th percentile of ADC_slow_ and certain overlap existed between groups existed, deprived of reflecting the realistic heterogeneity [33].

Moreover, it was because we separated the diffusion from perfusion through high b-values drastically that ADC_fast_ value (0.0428 ± 0.0741, *P *< 0.015) of malignant breast carcinoma was higher than benign tumor in our study. Meanwhile, most studies supported that abundant blood capillaries in tumor tissues might give a probable explanation that the high perfusion replenished the constrained liquid movement [34]. However, the f value (0.5940 ± 0.1401 vs. 0.4948 ± 0.1744, *P*<0.0060) of benign lesions was higher than malignancy, which was in contrast to the conventional assumption [35]. The possible reason was that on the one hand, the vessels from malignant tumors were oriented to small, leaky and few efficient tumor capillaries and tortuous vascular hyperplasia along with farraginous vascular branches in malignant lesion contributed to it [36]. On the other hand, it was indicated that the microvascular compression derived from the multitudinous cell density resulted in the lower f value [37]. The short TE acquisitions (TE<100 msec) in our study compared to the TE = 103 msec used by Sigmund et al. [38], as shown by Lemke et al. [39], might also result in the variability of low f value. Furthermore, Bokacheva L et al. had implied that lesions ROIs excluding the tumor edges conduced to lower perfusion fraction due to the tumor periphery were more vascularized than the red tumor center [40]. Some scholars also inferred that the change of ADC_fast_ was dependent on f, probably on account of the f signifying the blood-carrying capacity of the capillaries, but the ADC_fast_ reflecting the flow rate of blood, which changed when the microvascular diameter changed with volume invariability. Interestingly it could explain our data observed at length [41, 42].

We further reported the stretched exponential model that characterized the non-Gaussian behavior of molecular diffusion and reflected the degree of intravoxel heterogeneity of biological tissue for breast cancer. Bennnett et al. put forward a hypothesis that closer α to 1 and the higher homogeneity it would be [22]. In this study, α values of malignant lesions were lower than those of benign structures, for the sake of the fact that malignant lesions were associated with considerable histological heterogeneity. The high degree of cellular pleomorphism indicated high variability, the existence of intravoxel microscopic cystic or necrotic foci and even the heterogeneity in vascular structures [43, 44]. Besides, the DDC values were statistically higher in the malignant group while α exhibited lower in malignancy as compared with the benign group, which disagreed with the adopted evidence [45, 46]. The result suggested the inverse correlation between tumor cellularity and diffusion coefficient, indicating that the inhomogeneous necrosis and cystic components emerged in the target area. Thus, the negative relationship warranted further lager cohort validation, and histopathological correlation would comprehend the puzzle to some extent.

Additionally, AQP involved in cell migration for tumor angiogenesis and local invasion took charge of the water transport through membranes. When ultra-high b-values offered, the expression of AQP must be affected [26]. The ADC_AQP_ values (0.3636 ± 0.2175 vs 0.3699 ± 0.1139) of breast cancer were higher than the benign group, which was in support of the idea that AQP facilitated cell migration, not only relevant to angiogenesis but also to tumor spread, glial scarring, would healing and any other phenomena containing immune-cell chemotaxis [47]. Whereas the ADC_AQP_ showed no statistical differences in the current test. It would depend upon that the quantitative diffusion fitting model might be influenced by both target tissue and the choice of optimal b-value. Moreover, the inclusion of high b-values in our study prolonged the TE generating the consequent decrease in SNR. Previously published studies for preferable b value of breast lesion were still insufficient for all diffusion model, which needed to be optimized through in-depth research [46].

We considered that the comparison among the multiparametric utilization should establish criteria presenting a good differential diagnostic ability with not only the AUC greater than 0.75 but also outperforming any other quantitative parameters used in isolation. The required ROC analysis demonstrated that the ADC_avg_ still achieved good diagnostic efficacy (AUC = 0.806) with the highest specificity (97.56%) for breast cancer detections ranking only second to the multiparametric combination (ADC_avg-_ADC_fast_, AUC = 0.807). However, the excellent specificity was at the expense of decreased sensitivity for the sake of the reduction of false positive rate. Fornasa et al. [48] had reported fat necrosis exhibited malignant features like irregular mass and architectural distortion in accordance with our result. With regard to the combination of multiple variables, they might lead to a higher diagnostic power compared to the utilization of single parametric features. Surprisingly, the diagnostic efficacy of ADC_avg-_ADC_fast_ was the same as ADC_avg-_ADC_fast_-α (AUC = 0.807) in our study, signifying that the simplified model could also warrant the heterogeneity of malignant lesions. Furthermore, compared with other routine quantitative measurements, the highest Youden indices of ADC_avg-_ADC_fast_ with a sensitivity of 68.42% and specificity of 87.8% implied the superiority of differential diagnosis available in clinical practice, increasing its feasibility as a non-invasive tool.

There were several limitations to the current study. First, our investigation was constrained by relatively small sample size. The prospective validation of multiparametric combination model needed an overwhelming amount of data and develop a nomogram as supplement to present variables visually. Second, the stage of breast lesions was not respectively evaluated, so whether the lymph node metastases had existed was likely to become an influencing factor as Kamitani et al. [49] had observed that diffusion coefficients were higher in cases that were positive for axillary lymph node, and where micro-necrosis and fibrosis inside the lesion mattered. Third, neither age nor menstrual cycle were controlled in the enrollment. Forth, this developed model needed a further validation. Lastly, the choice of b-values was not optimized for all diffusion models. Generally, the b-values in our study were used to meet a wide range for diffusion. This study suggested that a comprehensive evaluation of breast cancer patients using advanced imaging may increase insight into tumor physiology. It was important to make the individualized treatment plans for high-risk women and such finding paved the way for deeper investigation of the potential of multiparametric MRI in the differentiation of breast cancer.

In conclusion, our research proved that multiple b-values diffusional exponential model contributed to the differential diagnosis of malignant breast lesions and multiparametric quantitative imaging with the combination of ADC_avg_ and ADC _fast_ could enhance the diagnostic ability of breast cancer detection rate by reflecting more biological characteristics of breast tissue and lesions. Therefore, if multiparametric technique combined with the utilization of ADC_avg-_ADC_fast_ was implemented into standard scanning protocol may have opportunity to maximize diagnostic accuracy while avoiding unnecessary breast biopsies.

## Data Availability

The datasets analyzed in this study are available from the corresponding author on request.
